# Sentiment analysis for measuring hope and fear from Reddit posts during the 2022 Russo-Ukrainian conflict

**DOI:** 10.3389/frai.2023.1163577

**Published:** 2023-04-05

**Authors:** Alessio Guerra, Oktay Karakuş

**Affiliations:** School of Computer Science and Informatics, Cardiff University, Cardiff, United Kingdom

**Keywords:** text mining (TM), social media, sentiment (SEN) analysis, hope, fear

## Abstract

This article proposes a novel lexicon-based unsupervised sentiment analysis method to measure the “*hope*” and “*fear*” for the 2022 Ukrainian-Russian Conflict. *Reddit.com* is utilized as the main source of human reactions to daily events during nearly the first 3 months of the conflict. The top 50 “hot” posts of six different subreddits about Ukraine and news (Ukraine, worldnews, Ukraina, UkrainianConflict, UkraineWarVideoReport, and UkraineWarReports) along with their relative comments are scraped every day between 10th of May and 28th of July, and a novel data set is created. On this corpus, multiple analyzes, such as (1) public interest, (2) Hope/Fear score, and (3) stock price interaction, are employed. We use a dictionary approach, which scores the hopefulness of every submitted user post. The Latent Dirichlet Allocation (LDA) algorithm of topic modeling is also utilized to understand the main issues raised by users and what are the key talking points. Experimental analysis shows that the hope strongly decreases after the symbolic and strategic losses of Azovstal (Mariupol) and Severodonetsk. Spikes in hope/fear, both positives and negatives, are present not only after important battles, but also after some non-military events, such as Eurovision and football games.

## 1. Introduction

For many years, the war in Europe has been only one of a dark memory. When on the 24th of February 2022, The Russian Federation declared war on Ukraine, the news came as a shock to most people around the world (Faiola, 2022). It was thought at that time that the presence of NATO and the European Union (EU) would be strong enough to guarantee peace in a short time. However unfortunately, peace was not restored due to the reason that both parties are neither part of NATO nor the EU, but they are both former members of the USSR, and the conflict is still going on even in early 2023.

In war, the morale of the nations is one of the most important aspects (Pope, 1941) since it is what pushes a country, most importantly, a country that keeps fighting. In the case of a country defending its own land, the morale does not only regard the two–belligerent country but mostly the defenders. In fact, at first, the Ukrainian chance for success has been seen as tied to the support of the Western countries (Galston, 2022), a need that was also confirmed by the Ukrainian president himself (France 24, 2022). For this reason, the feelings of the Western countries, which support Ukraine, could be a decisive factor in the future of the conflict. If the Western audience would perceive this conflict as a lost battle, which, if dragged on, would have bad repercussions on their daily life and only cause more harm to Ukrainians, then it could force them pressurize their governments into stopping the support given to Ukraine. On the other hand, if there is the hope of winning the conflict, then it is possible for the governments to keep guaranteeing active support to Ukraine and impose costly sanctions on Russia.

According to the Collins dictionary, hope is an uncountable noun and is described as “a feeling of desire and expectation that things will go well in the future” (Collins Dictionary, 2022b). Conversely, fear is defined as “a thought that something unpleasant might happen or might have happened” (Collins Dictionary, 2022a). As grammatical objects they may be uncountable nouns, however, the main purpose of this article was to promote various text mining and sentiment analysis techniques to measure “*Hope*” and its negative counterpart “*Fear*” using social media posts from Reddit.com–the social news aggregation, content rating, and discussion website.

## 2. Background and related works

From a general point of view, “sentiment analysis” can be defined as the procedure of utilizing important techniques, such as natural language processing, text analysis, and mining, to extract and interpret subjective and human-related information (Medhat et al., 2014). The source of information for sentiment analysis can be diverse, e.g., written text or voice, whilst the entities could be events, topics, individuals, and many more (Liu, 2020). Sentiment analysis is also a broader name for many other tasks, such as opinion mining, sentiment mining, emotion analysis, and mining (Dave et al., 2003; Nasukawa and Yi, 2003; Liu, 2020). Text data mining can be defined as the process of extracting information from data sources that are mainly made of text (Hearst, 1999). Text mining can be utilized for different purposes and with many techniques such as topic modeling (Rehurek and Sojka, 2010) and sentiment analysis (Feldman, 2013). Text-related sentiment analysis is a versatile approach that helps to automatically extract meaningful information from the written text and is useful to pursue many different objectives, such as assessing and monitoring psychological disorders (Zucco et al., 2017), to evaluate human behaviors during the football 2014 FIFA World Cup (Yu and Wang, 2015), to detect emotions in general (Peng et al., 2021) or to use them to conclude on gender differences (Thelwall et al., 2010), or even to make predictions on the stock market (Pagolu et al., 2016) and measure the heterogeneity of investors *via* their social media posts (Ji and Han, 2022).

Considering the large number of social networks that are recently continuing to expand with regard to the number of users, and are capable of reaching more audiences from nearly all levels of the community, social media has naturally become the main source of information for text mining and sentiment analysis purposes. Sentiment analysis has been used to interpret data from different social network sources, the most obvious example of which is the Twitter (Hu et al., 2013; Yu and Wang, 2015; Giachanou and Crestani, 2016; Ji and Han, 2022). In addition, other popular social networks have also been used as the data source for sentiment analysis-related purposes, e.g., Facebook (Ortigosa et al., 2014), Reddit (Melton et al., 2021), Myspace (Thelwall et al., 2010), and even YouTube comments (Tripto and Ali, 2018).

Despite the social media being one of the most common sources of data, sentiment analysis has also found an application basis for many more text corpora–to name but a few: movie (Thet et al., 2010) or product reviews (Haque et al., 2018), newspaper articles (Balahur and Steinberger, 2009), or emails (Liu and Lee, 2018). Many of the analyzes mentioned previously mostly focus on understanding if a text is positive, negative, or neutral as a classifier (Pak and Paroubek, 2010), and/or promoting the utilization of various scoring systems (Naldi, 2019). It is also possible to employ similar analyzes to understand if a text utilizes subjective or objective language (Liu, 2010) or to interpret which emotions are conveyed (Yadollahi et al., 2017).

Having a vast amount of data containing a multitude of types of human emotions is not only highly exciting in terms of computational data analysis research, but it is also seen to be useful for human behavioral research. In general, there are two main theories on how emotions are formed in the human brain. The first is the discrete emotion theory that says emotions arise from separate neural systems (Shaver et al., 1987; Ekman et al., 2013). In these seminal studies, Ekman et al. (2013) recognize six basic emotions of anger, disgust, fear, joy, sadness, and surprise, whilst Shaver et al. (1987) recognize anger, fear, joy, love, sadness, and surprise. On the other hand, the dimensional model says that a common and interconnected neurophysiological system causes all effective states (Lövheim, 2012; Plutchik and Kellerman, 2013). In particular, Plutchik and Kellerman (2013) recognize anger, anticipation, disgust, fear, joy, sadness, surprise, and trust, whilst (Lövheim, 2012) recognizes anger, disgust, distress, fear, joy, interest, shame, and surprise. Creating statistical correlation and independence analysis approaches are also highly important to provide evidence for the aforementioned human behavioral studies.

This article aims to develop a novel lexicon-based unsupervised method for the purpose of measuring the “hope” and “fear” during the 2022 Ukrainian–Russian Conflict. As the source of human reactions, we utilize the social media platform–Reddit.com–to collect daily posts during nearly the first 3 months of the conflict. The structure of this social network–Reddit.com–allows for discussing specific topics (in Reddit terminology “posting in specific *subreddits*”), without short limitations in the number of characters that can be posted. This approach makes it easy to mine for opinions about the Ukrainian conflict, to get an idea of what people think about it, and how hopeful/fearful they are. To achieve this goal, the top 50 “hot” posts of six different subreddits about Ukraine and news (Ukraine, worldnews, Ukraina, UkrainianConflict, UkraineWarVideoReport, and UkraineWarReports) and their relative comments are scraped to create a novel data set. We employed various important analyzes on this corpus to promote the use of a dictionary approach, which scores the hopefulness of every submitted user post. Finally, we performed a topic modeling analysis using the Latent Dirichlet Allocation (LDA) algorithm to understand the main issues that are raised by users and what are the key talking points.

This research aimed to fill the gap present in the literature regarding opinion mining, specifically for *hope*. The main analysis consists of mapping hope measured with the newly proposed method *via* developing a “hope dictionary.” In particular, first, the trend of hope over time is monitored. It is later compared with some of the most important events that happened during the study time frame. This approach ascertains how such events influenced the public perception of the conflict and provides evidence about the validity of the proposed hope measure. Fear is measured *via* the same dictionary approach and mapped over the same study time period using the National Research Council (NRC) Word-Emotion Association (Mohammad and Turney, 2013) “fear” dictionary. Furthermore, individual topics extracted *via* the topic modeling observations are studied to interpret whether there is a correlation with “hope/fear” and what kind of relationship they present if this were the case. Sentiment analysis is also employed to track the popularity of individual leaders (Putin and Zelensky) and the Russian and Ukrainian governments. Finally, stocks, such as Gazprom and indices (gas prices and Russian and Ukrainian bonds), are analyzed to interpret whether there is a relationship between the developed hope score and the stock market.

## 3. Methodology

### 3.1. Reddit data

Reddit has been chosen since its structure allows easy group submissions about a specific topic. Reddit is known to be different from other social media platforms, such as Twitter, since it is based on communities (i.e. subreddits) rather than people, hence, the success of the content is less influenced by the success of the author. Anonymity is an important aspect of Reddit therefore it creates a forum with social media aspects. To gather data for the analysis, it was necessary to obtain them from Reddit. The best way to achieve this goal is to use the official Reddit API. To do so, it is necessary to register as a developer on their website, authenticate, register the app, and state its purpose and functionality. Once the said procedure is completed, the developer can request for a token, which has to be specified along with the client id, user agent, username, and password every time new data are requested.

Six subreddits were chosen for their relevance to the conflict:

- r/Ukraine
- r/worldnews
- r/ukraina
- r/UkrainianConflict
- r/UkraineWarVideoReport
- r/UkraineWarReports.

The script developed in Python crawls the top 50 posts for each subreddit and the relative comments. Subsequently, it combines the newly gathered submissions with the previously collected ones. It then removes eventual duplicates using the submission id. For every submission, the subsequent information was obtained:

- title (only for posts): the title of the post
- text: the actual content of submission
- upvotes (a method by which users can show their approval/support for a post)
- author
- date
- id: the unique submission id
- flair: categorization of the post by the author
- type: post or comment
- parent_id
- subreddit.

The data collection process started on the 10th of May 2022 and was completed on the 28th of July 2022. It was conducted daily around 3.00 pm UK time. More than 1.2 million unique observations were gathered within this time frame. All the data sets developed for the purposes of this article are summarized in Table A1 in Appendix 1.

### 3.2. Pre-processing stages

The data obtained through the collection process were not useful on their own. They had to be processed to be analyzed and explored. First, some cleaning had to be done. Not all the observations gathered were useful. In fact, some of the submissions in the r/worldnews subreddit were not about the conflict. To eliminate the irrelevant submissions, only those posts with the flair “Ukraine/Russia” had to be kept. The only issue is that flair is assigned only to “post” type submissions, but not to comments.

Luckily, the structure of Reddit allows us to use id and parent_id to move upwards to the original post from every comment. Every comment is like a tree branch in a forest-like structure, with every post representing a single tree. Due to this principle, it was possible to extract the “ancestor_id” of every submission and use it to assign a flair to the comments. This allowed us to identify and remove the submissions without the relevant flair from the r/worldnews subreddit.

The next step would be to convert all the words in each post to lowercase. Subsequently, we obtained the score for a specific emotion for every submission. To reach this goal, the number of words related to the investigated emotion in every entry was counted.

Another useful information to be extracted is the polarity score. Using a different sentiment analysis approach, the “text” of a post or comment would receive a score that ranges from –1 to 1 according to its sentiment. A score of –1 indicates a very negative meaning, whilst 1 indicates a very positive one. The score was extracted using the *sentiment.polarity* method from the *TextBlob* python module. Another method, *sentiment.subjectivity*, from the same module was also used that allows us to understand if the author is stating facts or if they are voicing an opinion. Subjectivity ranges from a score of 0, which indicates a very subjective text, to 1, which indicates a very objective one.

One of the problems with the dictionary-based sentiment analysis is that it arbitrarily favors long texts. In fact, with a higher word count, there are more chances of finding relevant words. Furthermore, it increases the score cap for a submission. A one-word comment could have a maximum score of 1, whilst a hundred-word comment could potentially score 100. To solve this issue, a new parameter called “*w*_*lenght*_” was created. It stores the emotion score (*N*_*emotion*_) divided by the length of the submission (*length*) multiplied by 100 and can be expressed as


(1) $$\begin{array}{l} w_{lenght}=\frac{N_{emotion}}{length\times 100}. \end{array}$$

Another improvement to be made regarded the weight of singular opinions. There are opinions which are more popular than others. On Reddit, it is easy to understand whether one post is popular or not by looking at the number of upvotes. To have a better understanding of the public opinion, it was relevant to weigh the hope score with the number of upvotes. Whilst being an improvement, simply multiplying the *w*_*lenght*_ score in Equation (1) for the number of upvotes would disfavor popular comments in unpopular posts. A very successful post would have a very high number of visualizations, comments, and upvotes. A comment X, viewed by 100 people and upvoted by 10 (10%), would have a higher score than a comment Y, viewed by 10 people and upvoted by 5 (50% of viewers). To solve this issue, the number of upvotes needed to be weighted on the number of comments on a post, to obtain its relative popularity (as opposed to the absolute one). A parameter storing the number of comments for every post (“*sub*_*in*−*post*_”) was obtained by counting the submissions for every “ancestor_id.”

Finally, another parameter “*w*_*upvotes*_” was created. It stores the value that “*w*_*lenght*_” multiplied by the number of upvotes divided by “*sub*_*in*−*post*_” and can be expressed as


(2) $$\begin{array}{l} w_{upvotes}=\frac{w_{length}\times upvotes}{sub_{in-post}}. \end{array}$$

**Figure 1:**
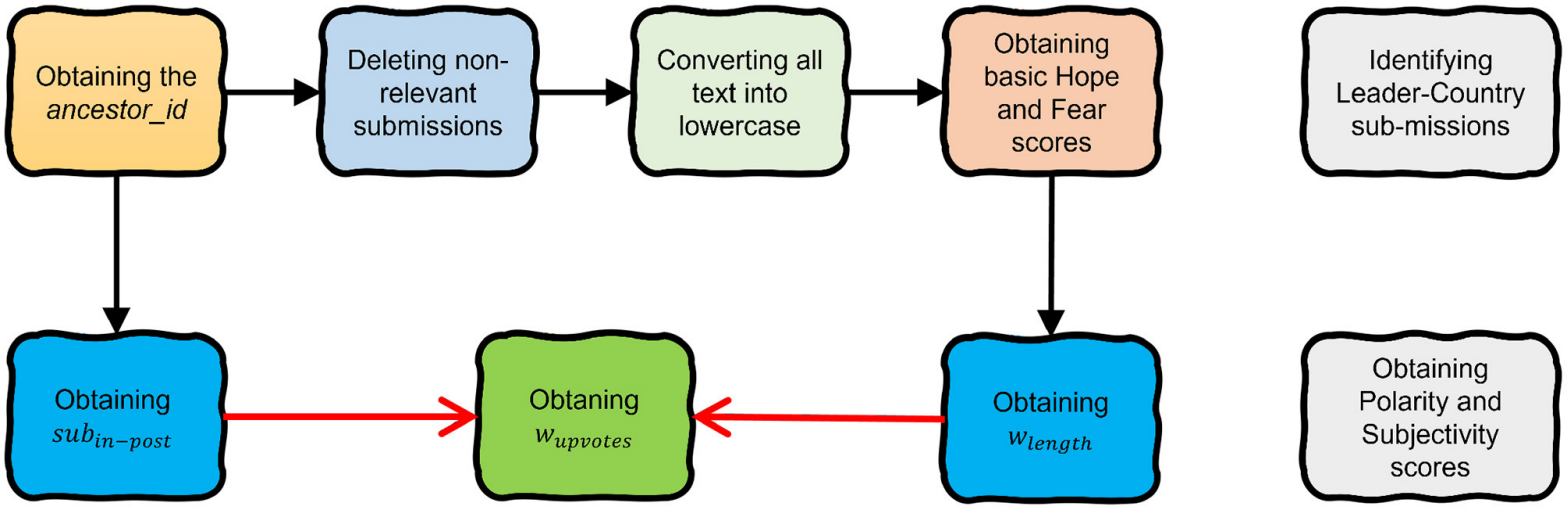
The pre-processing workflow shows the stages of obtaining the emotion of *w*_*upvotes*_. The two gray blocks on the right show additional pre-processing stages required for the experimental analyzes in this article.

Hence, *w*_*upvotes*_ becomes the emotion score that is weighted on its length, the upvotes, and the relative popularity. The flow diagram of the general pre-processing process is depicted in Figure 1.

### 3.3. Measuring hope and fear

Overall interest in the conflict has been measured in two different ways: (i) the number of submissions and (ii) the popularity of the posts. For the former, data were grouped by each day, and the number of daily submissions was counted. This includes both posts and comments, giving a good idea of the engagement trend. The latter studies the daily average number of upvotes for each post. Comments were excluded since a popular post is likely to host many comments with just one upvote, which would significantly lower the average. To achieve this goal, a post-only database was created. Data were grouped by date and the mean value for upvotes was computed.

Complementing the aforementioned second method with the first one is very useful to give a proper idea of the general interest trend. The number of posts could have been influenced by a small number of users who are somewhat involved with the conflict, whilst the public might not be that interested. This development can be tested by looking at the popularity of the posts. In fact, popular posts have many upvotes. To reach them, submission needs to have the likeness or the attention of a big group of users.

The main goal of this study was to map hope in Western public opinion for the Russo-Ukrainian war. There is a gap in the literature regarding this specific issue. There is, indeed, no scholarly accepted way to automatically measure hope.

There are many ways to tackle sentiment analysis, such as machine learning or dictionary-based approaches. The first one would have required labeling a data set, saying what is hopeful and what is not. To properly do that, linguistic expertise is a requirement. On the other side, using a dictionary-based approach would allow using scholarly accepted dictionaries. Hence, this article concerns a dictionary-based approach.

Two issues had to be addressed to complete a dictionary-based analysis: that are linguistic and technical ones. At this point, we ask several important questions: What is hope and how do we measure it? According to the Collins dictionary, “Hope is a feeling of desire and expectation that things will go well in the future.” Picking apart this definition helps to understand what are the elements that construct hope. The keywords are “feeling,” “well,” and “expectation in the future.” A feeling is something inherently subjective to the person who feels them. Well, in this case, indicates a positive outcome. The expectation is “something looked forward to, whether feared or hoped for” and it is a synonym for anticipation.

Since there is no “hope” dictionary to the best of our knowledge, one had to be developed. As a starting point, the NRC sentiment and emotion lexicon was used. The NRC Emotion Lexicon is a list of English words and their associations with eight basic emotions (anger, fear, anticipation, trust, surprise, sadness, joy, and disgust) and two sentiments (negative and positive). The annotations were manually done by crowdsourcing. As mentioned above, among the emotions cataloged in this dictionary, there are three emotions–“anticipation,” “positive,” and “joy”–that require a careful analysis. According to the Collins Dictionary, the definition of hope given earlier, *something to be hopeful*, needs to be *subjective anticipation of a positive outcome*. Hence, the three dictionaries of “anticipation,” “positive,” and “joy” were cross-referenced to find the words that showed “anticipation” and at least one between “positive” and “joy.”

Due to this procedure, a “hope” dictionary is developed. The lexicon respects two of the three parameters: “anticipation” and “positive outcome.” To satisfy the third parameter, all the Reddit submissions were analyzed through the *textblob.subjectivity* function. It gives a score that ranges from 0 (not subjective) to 1 (very subjective). For the third parameter, only the submissions that present a minimum score of 0.5 were analyzed.

Once the dictionary was developed, it needed to be implemented. Every submission is characterized by a “text” column that contains the message sent by the user. The script counts how many times words present in the “hope” dictionary are also present in the “text.” In this way, a raw hope score, notated as *hope*_*score*_, is obtained, which is the refined version of *w*_*upvotes*_ in Equation (2) for the hope analysis. The raw hope score can be calculated as


(3) $$\begin{array}{l} hope_{score}=\frac{\frac{N_{hope}}{length\times 100}\times upvotes}{sub_{in-post}}. \end{array}$$

Fear was measured in the same way as hope in Equation (3). It is dictionary based and the score is obtained by counting the fear-related words in every submission. The utilized dictionary was the same NRC one that is used to obtain “anticipation,” “joy,” and “positive” words. The *fear*_*score*_ is calculated as


(4) $$\begin{array}{l} fear_{score}=\frac{\frac{N_{fear}}{length\times 100}\times upvotes}{sub_{in-post}} \end{array}$$

### 3.4. Leader and country analysis

To obtain the Leader analysis data, two new databases were created. The first one had only the submission containing the name “Zelenskyy” or its variations “Zelens'kyj” or “Zelensky.” The second one, instead, included only observations that presented the name “Putin.” Differently from the other analysis, hope and fear were not analyzed, but the focus was on the sentiment polarity score. The polarity method from TextBlob was employed. It gives a score that ranges from –1 to 1, with the former representing a negative opinion, whilst the latter showed a positive one. After both databases were grouped by day, the mean daily polarity score was computed.

Similar to the Zelenskyy vs. Putin analysis, two new databases were created. The first one included only submissions that contained the name “Ukraine,” whilst the second only had only observations that presented the name “Russia.” Subsequently, the polarity score was measured using the TextBlob polarity method. Then, observations were grouped by day and the daily average polarity score was computed.

### 3.5. Stock market analysis

After collecting historical prices on six different stocks and financial titles (UK oil and gas, Russian ruble and US dollar exchange rate, the price of gas, and the price of crude oil), they were added to the “daily” database. The said database contains the weighted average daily value for hope and fear.

We developed a linear regression model having the price of the ticker as the dependent variable and either the average weighted daily hope score or the weighted average daily fear score as the independent one. Then for each data set, we ran this linear regression model and calculated the corresponding parameters for each modeling.

### 3.6. Topic modeling

The aim of this study was to understand what the gathered submissions are about, through topic modeling. Topic modeling is an unsupervised machine learning technique that allows us to organize, understand, and summarize large bodies of text. It can be described as a method for extracting meaning out of the textual data by extracting groups of words, or abstract topics, from a collection of documents that best represent the information in the collection. More specifically, this technique returns a probabilistic distribution of different topics of discussion, where each topic is associated with a given document by a certain likelihood score. A document could contain different topics at the same time in different proportions.

We first created a corpus and dropped less frequent terms in it. Now that the text data have been processed, the optimal number of topics (*K*) is estimated. Using the *searchK()* function, the different distributions of *K* (from 2 to 10) are elaborated, so that it is possible to interpret the results and make a guess on the optimal number of topics in the model. To find the optimal number of topics, it is necessary to plot the distributions of K topics discovered according to various goodness-of-fit measures such as semantic coherence and exclusivity. Semantic coherence measures the frequency in which the most probable words in each topic occur together within the same document. Exclusivity, on the other hand, checks the extent to which the top words for a topic are not top words in other topics. Coherence measures how a topic is strongly present and identifiable in documents, whilst exclusivity measures how much the topic differs from each other. The goal is to maximize both, whilst keeping the likelihood high and residuals low enough. Then, the distribution of the topics in the document is examined to see if there is a prominence of one topic over the others or if they have similar distributions (bad sign). Subsequently, a word cloud for every topic is created. It shows in a graphical cloud all the top words, with size changing according to the relative frequency of the words. Using the *labelTopics()* function, the words that are classified into topics to better read and interpret them are inspected. This function generates a group of words that summarize each topic and measure the associations between keywords and topics. The most representative documents for each topic are then extracted. This is useful because it helps us to give a more concrete idea of what each topic is about, using a real review as an example. The relationship between metadata and topics is studied. It is carried out by defining the correlation model by applying the *estimateEffect()* function. This function performs a regression that returns the topic proportions as the outcome variable. The output of the function aimed to demonstrate the effect of the covariates of the topics. To conclude, the correlation between topics is studied.

## 4. Experimental analysis

### 4.1. Hope–fear analysis

**Figure 2:**
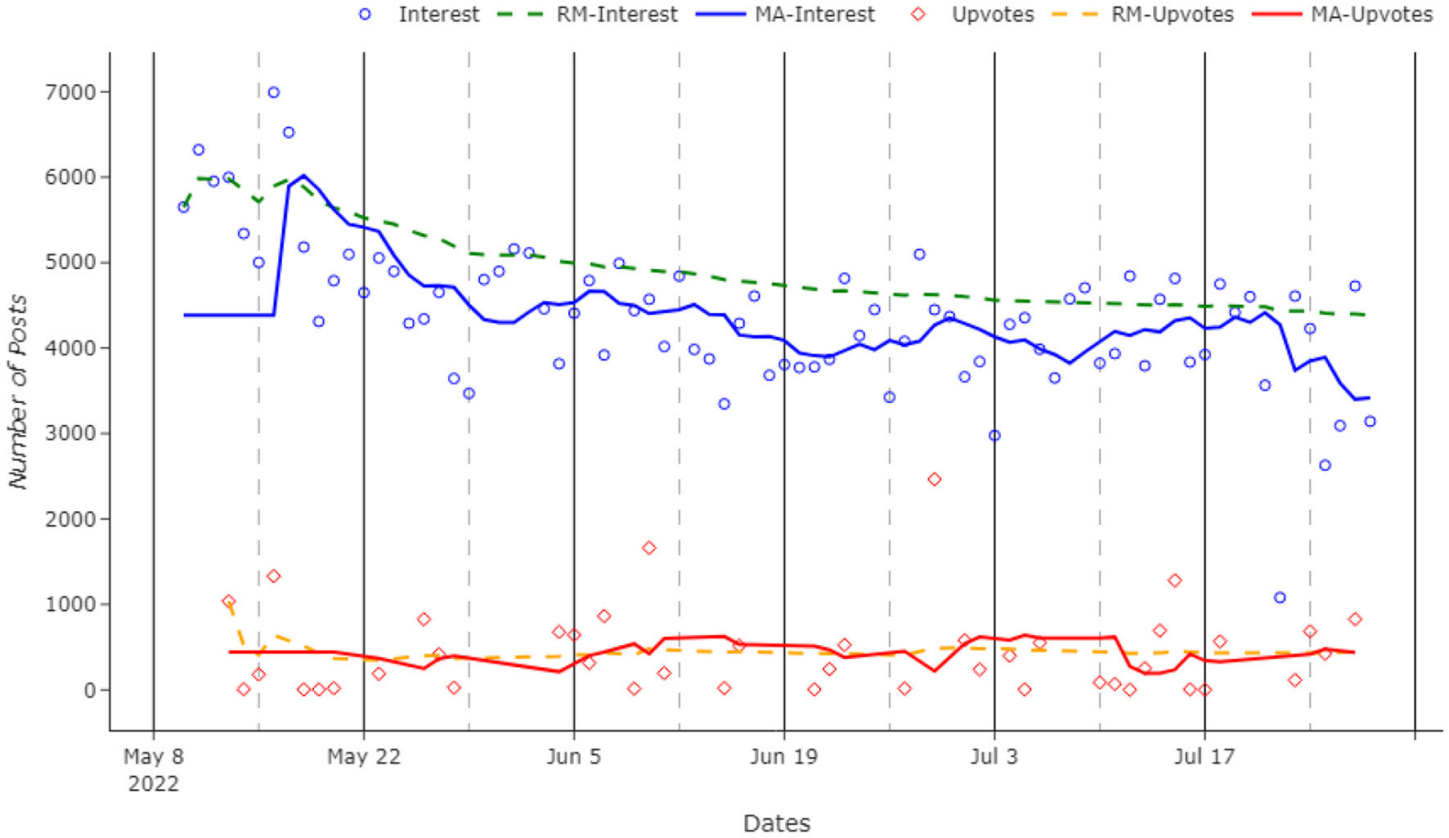
Number of submissions and daily average number of upvotes over time. The acronyms in the figure legend are: RM stands for the running mean, whilst MA is the moving average.

Our Hope–Fear analysis starts by measuring the public interest in the war and their intention to share posts on social media, as shown in Figure 2. Overall social media interest during the conflict has been slowly but steadily decreasing for the whole analyzed time window. With an average of 4,335 daily submissions, in the first few days, there were plenty of submissions, with a peak of 6,993 posts in one single day on the 16th of May 2022. In the last part of the explored time, the numbers became lower, with a negative peak of only 1,080 submissions in 1 day on the 22nd of July 2022, 5,919 less than its maximum.

When we evaluate the daily upvote rates in Figure 2, differently from the aforementioned analysis, there are no significant changes in the trend in the number of upvotes over time. The daily average itself is very volatile, but the trend remains stable. This could mean that, whilst the users are still receptive and supportive toward the Ukrainian conflict (they keep upvoting the most important posts), they are less engaged, posting and commenting less.

**Figure 3:**
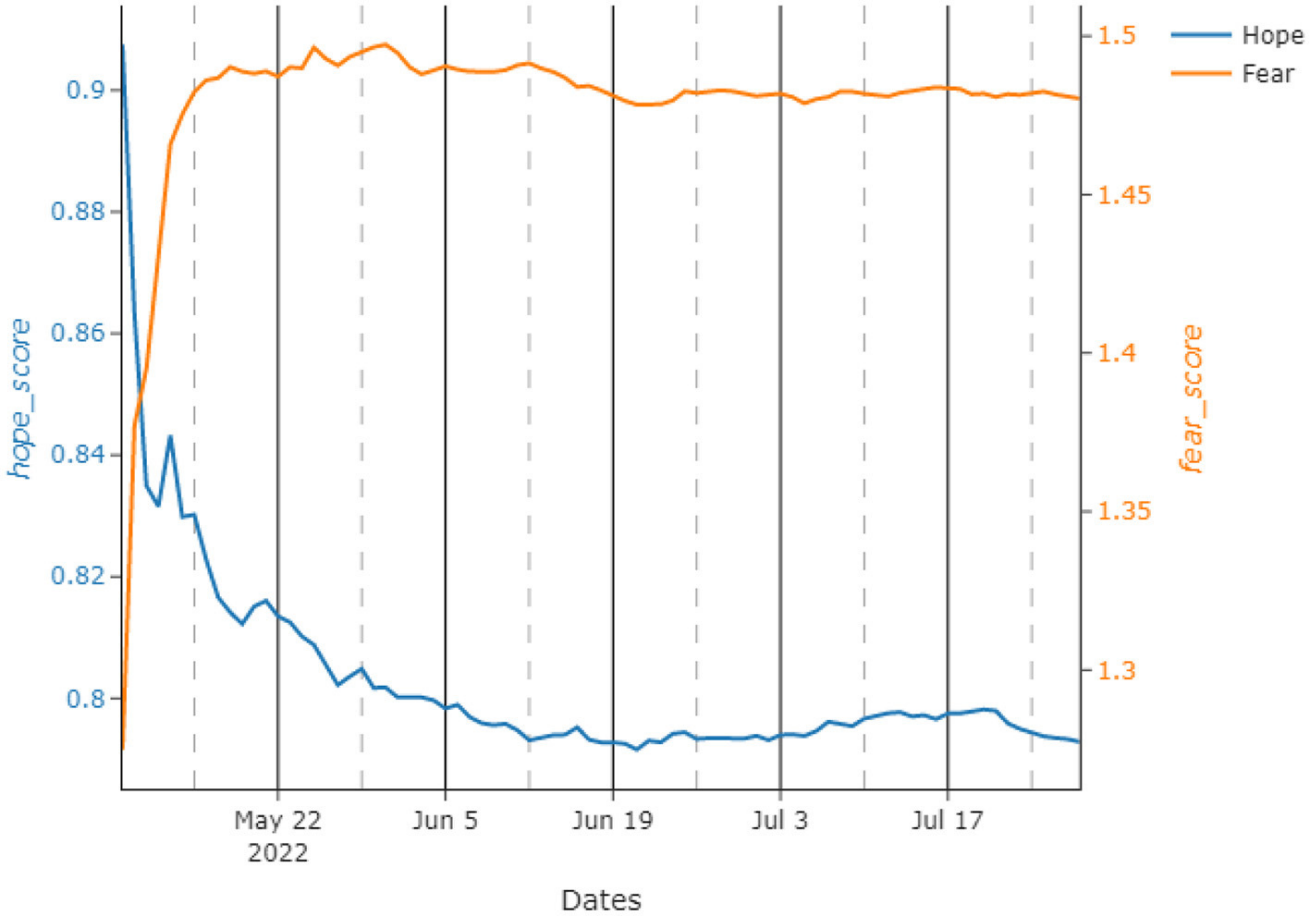
Running means for the proposed hope and fear scores during the test interval between May and July 2022. The left *y*-axis refers to *hope*_*score*_, whilst the right one is for *fear*_*score*_.

Due to this steady trend in upvotes and the number of posts each day, we calculated the daily hope score by using the expression given in Equation (3). As it is possible to observe from the graph given in Figure 3, the hope score during the analyzed time period is decreasing and finds a nearly steady state after half of the observed period in terms of its running mean visualization. After the initially big drop, the score seems to stabilize at a lower value. This trend seems to reflect what happens during the war. In fact, the big drop happens around the fall of Azovstal (Mariupol) and Severodonetsk. Successively, it mirrors the “phase two” of the Russian offensive, with a slow and steady trend of hope score. This aspect is also reflected by the fact that central 50% of the observations of the hope score is in the range of 0.054, whilst the total range is 0.264, as it is possible to see from the descriptive statistics in Table 1.

**Table 1:** Descriptive statistics for the whole analysis.

	**Interest**	**Upvotes**	**Hope**	**Fear**	**Zelenskyy**	**Putin**	**Ukraine**	**Russia**
Days	80	43	81	81	81	81	81	81
Mean	4383.06	441.38	0.7928	1.4803	0.0949	0.0381	0.0901	0.0402
St. Deviation	861.06	518.90	0.0425	0.0591	0.0417	0.0162	0.0129	0.0108
Minimum	1080.00	1.00	0.6434	1.2749	–0.0311	–0.0053	0.0627	0.0067
25*th* percentile	3840.50	20.00	0.7650	1.4405	0.0716	0.0254	0.0813	0.0359
50*th* percentile	4412.50	253.00	0.7926	1.4848	0.0959	0.0390	0.0896	0.0417
75*th* percentile	4815.25	659.50	0.8189	1.5213	0.1206	0.0476	0.0991	0.0480
Maximum	6993.00	2464.67	0.9075	1.6199	0.2138	0.0728	0.1210	0.0596

Similarly, by using the expression developed in Equation (4), we calculated the fear score for the same time period. Despite being pretty volatile, fear remains stable for the whole analysis just after initial couple of days. This is an interesting observation, especially when compared to hope, which decreases in the same time period. Hope–Fear results are slightly negatively correlated, with a Pearson's correlation index of –0.986. Here, to clearly interpret this phenomenon, we plot the running means of Hope and Fear on the same axes in Figure 3.

### 4.2. Validation of hope/fear scores

To validate and better visualize the proposed hope/fear scores, we investigated 18 important events within the experimental period. To reach this goal, observations were grouped by day and the mean hope score was computed. The overall mean of the hope score was also calculated and a new column that contained the overall mean–each day's average was created. The said important events chosen for the validation analysis are given below:

1. **May 9** - Failed Russian Donetsk River crossing. Ukrainian sources declare that during the crossing, 70 heavy Russian units were destroyed or lost.
2. **May 13** - American–Russian talks. Lloyd Austin (American secretary of defense) and Sergei Shoigu (Russian minister of defense) held telephoneic talks for the first time since the start of the invasion.
3. **May 15** - Ukraine won the Eurovision 2022 Song Contest, on account of to an overwhelming popular vote. Stefania performed by the Kalash Orchestra won with 192 votes from the jury (4th place) and 439 from the televote. The second place went to the United Kingdom with 466 total votes.
4. **May 17** - Azovstal, the steel factory of Mariupol, was lost. It was the last stand of the Azov Battalion, a controversial group, which contained many of the best-trained Ukrainian soldiers. This deprived Ukraine of a strategically important port and many soldiers, and allowed the Russians to unify the front.
5. **May 27** - Ninety percent of Severodonetsk was destroyed. The city is of big strategic importance since it could allow the Russians to encircle many Ukrainian units in Donbas.
6. **May 29** - The first visit of Zelenskyy outside of Kiev. This visit had the purpose to show that the president was not afraid of Russia taking him out.
7. **May 30** - Russian troops entered Severodonetsk.
8. **June 5** - Ukraine was eliminated of the World Cup 2022 qualifiers, after losing 1–0 to Wales, with a goal scored by Gareth Bale.
9. **June 12** - Ukrainian supplies and planes destroyed.
10. **June 16** - Sinking of a Russian ship. The Spasatel Vasily Bekh tug was sunk near Snake Island in the Black Sea.
11. **June 17** - Putin's speech at an economic forum in St. Petersburg.
12. **June 22** - Ukrainian drone strike on a Russian oil refinery.
13. **June 26** - Fourteen missiles hit Kyiv, damaging several buildings and a kindergarten.
14. **July 6** - Russian Duma prepared to go into a war economy, which would allow ordering companies to produce war supplies and make workers work overtime.
15. **July 7** - Zelenskyy gave a speech on the effectiveness of Western artillery. Furthermore, a technical pause from the Russian offensive started, with the aim to regroup.
16. **July 14** - The start of the volunteer mobilization, which required by the end of the month, 85 federal areas to recruit 400 men each.
17. **July 16** - The US House of Representatives approved a bipartisan bill that would grant $100 million in funds to train Ukrainian pilots to fly US fighter jets.
18. **July 23** - Four Kalibr missiles hit Odessa. Of those four, two were intercepted. The other two according to Russian sources destroyed a warship and a warehouse containing missiles.

**Figure 4:**
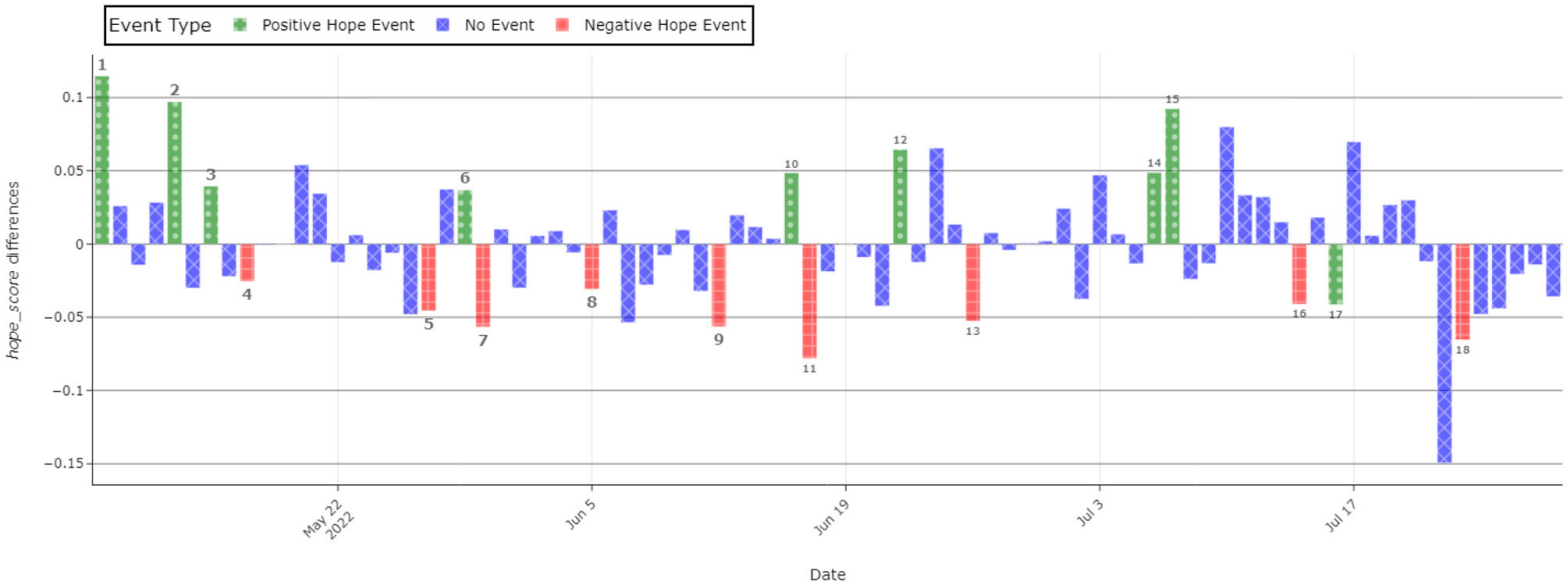
Deviation from the average hope. Each bar refers to the hope difference from the mean value. Numbers located above the bars correspond to the important events mentioned in Section 4.2.

The graph in Figure 4 shows how much above, or below, average hope scored during the analyzed period. Many of the spikes, both negative and positive, coincide with real-world events, which had an impact on the war or on the morale of Western public opinion. Some of the positive events include but are not limited to: the Ukrainian victory in the Eurovision contest (3), financial help packages from the United States (17), and the sinking of Russian ships (10). Negative ones include but are not limited to the loss of Azovstal (4), the fall of Severodonetsk (5), and the elimination of Ukraine from the World Cup 2022 (8).

As it is possible to observe in Figure 4, most of the biggest positive spikes are concentrated in the first few days, when phase 2 of the war had recently started. After the fall of Azovstal and Severodonetsk, a slower and more intense phase of the war starts. Russians advance slowly but steadily. This is also reflected in the graph, where we can observe few spikes and many observations being below average for the whole duration of June. In July, there was more movement, in fact, the United States developed a plan of military and financial aid to Ukraine. Furthermore, Turkey managed to broker a trade deal between Ukraine and Russia, which would allow Ukraine to export grain, avoiding famine in many countries (mainly in Africa). At the same time, Russian advance keeps proceeding recklessly, as shown by the negative spikes at the end of the month.

### 4.3. Country–leader analysis

**Figure 5:**
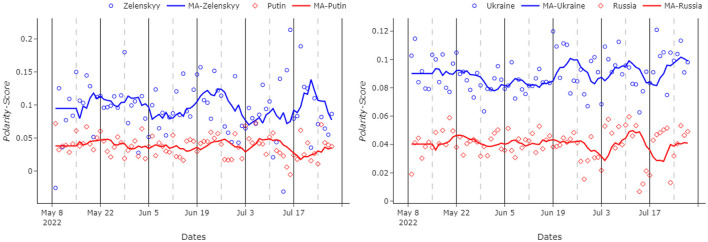
**(Left)** Polarity score for the two leaders. **(Right)** Polarity score for the two countries. MA graphs for each figure refer to the 7-day moving average of the original data. Zelenskyy/Putin and Ukraine/Russia polarity scores are represented with different markers, as shown in the figure legends.

In this case of the experiments, we try to measure public interest in countries (Ukraine-Russia) and leaders (Zelenskyy-Putin). As previously stated, the metric for popularity refers to the sentiment “polarity.” The first and most obvious consideration that emerges from this analysis presented in Figure 5 is that Zelenskyy, the president of Ukraine, presents a higher sentiment than Putin, the president of Russia. As it is possible to notice, Zelenskyy is consistently more popular than his Russian counterpart, for the whole analyzed period. In fact, the average polarity score for the Ukrainian president is 0.097, 2.6 times more than Putin, who scores a mere 0.037. Despite being less popular, the Russian president is more interesting to the Reddit community than Zelenskyy. In fact, his name is cited 30,663 times in the database, 7.2 times more than his Ukrainian counterpart, who is cited only 4,055 times.

Another interesting point is that, despite being relatively volatile, the trend seems to be consistent during the analyzed period. None of the two leaders presents an increase, or a decrease, in popularity. Zelenskyy shows higher volatility than Putin, but this is likely attributable to the smaller sample size.

The small sample size also causes big outliers in the Zelenskyy graph. For example, on the 14th of July 2022, the Ukrainian president showed a polarity score of –0.31, 0.128 below the average score. There were only 49 submissions naming Zelenskyy on that day. One of the first ones accused the president to be a Nazi and to have violated human rights in Donbas. Many comments answered these accusations defending the president. Saying for example:

“this is such a massive false equivalence. periodically i bother responding to it. here is my copy-paste nobody ever wants to engage with. non-extensive list examples of ways in which i think it's possible to differentiate the two cases:* zelensky has never used chemical weapons to suppress a revolt against his rule by an ethnic minority, * the us did not execute civilians en mass in any captured town [...]”

or:

“*this is a ludicrous comparison. whilst i don't agree with what the west did in iraq in early 2000's ….sadam hussein was committing genocide against the kurds, systematically slaughtering hundreds of thousands of people because of their race/religion. zelensky is not doing this, he is a democratically elected official and ukraine are a peaceful nation. so the idea that we (the west) are not allowed to comment on the russian invasion of ukraine because we've done something similar is lazy, ridiculous and without being rude to you, a tad stupid*.”

Most of those comments are saying that Zelenskyy and Ukraine did not commit atrocities, as affirmed by someone else. But (as it is later explained in the limitation part), many words with a negative sentiment, such as “suppress,” “execute,” “genocide,” “slaughtering,” “lazy,” and “stupid,” are used and the context is not interpreted. Having a big sample prevents these context-based exceptions from happening. For this specific day, the sample size is relatively small and is not able to counterbalance this single thread.

Another interesting insight is that there is no correlation between the popularity of Zelenskyy and Putin. The Pearson correlation index, in fact, is –0.03. It could have been possible to hypothesize a negative correlation between the two, maybe connected to the tides of the war. For example, if Russia was making gains Putin's popularity could be increasing, whilst Zelenskyy's would be decreasing. But this hypothesis is disproven by the evaluated data in the given time period. This could be explained by the fact that it is possible that Putin's popularity would not increase with a successful war since he has mostly been seen as the enemy.

Similar to the Putin vs. Zelenskyy analysis mentioned previously, it can be seen from Figure 5 that Ukraine scores evidently better than Russia. In fact, the former has scored consistently more than the latter with an average polarity score of 0.077, compared to an average polarity score of 0.044. In the same fashion as mentioned in the previous analysis, Russia is cited way more frequently than Ukraine. In fact, Russia is cited 137,419 times, whilst Ukraine is cited 89,736 times. Despite five of the six analyzed subreddits being named after Ukraine, the aforementioned result is found to be rather interesting since the real focus is on Russia.

The two trends seem to be very similar. In fact, the Pearson correlation index is 0.55. This similarity might be possible because the two countries are very often cited in the same submission, hence presenting identical polarity scores. To solve this issue, two new databases, which, respectively, contained “Ukraine” but not “Russia” and vice versa, are created. In this process, 33,790 observations for each database were dropped, removing more than one third of the original “Ukraine” database.

The new numbers highlight even more focus on Russia, which now counts almost double the number of citations than Ukraine, counting 103,629 against 55,946. The new data show an increase in the gap between the two countries. In fact, Ukraine, with an average score of 0.09, scores more than double that of Russia, which decreases its polarity score to 0.04. As expected, the Pearson correlation index also decreases significantly to 0.26, which remains still surprisingly high.

### 4.4. Stock market analysis

Four different tickers, regarding four different aspects connected to the war, were chosen: (1) United Kingdom Oil and gas stock price, (2) Ruble - US dollar exchange rate, (3) Oil price, and (4) Gas price. In particular, the most influential one pertains to gas prices, which have been used as leverage for a good portion of the conflict. Many Western countries, including but not limited to Italy and Germany, provide weapons and support to Ukraine but used to rely heavily on Russian gas for their energy needs. Russia has maneuvered the gas price and supply (for example, closing the gas pipeline Nord Stream 1) to try to weaken the support for the Ukrainians and lift the sanctions imposed on Russians. Furthermore, through the increase in gas prices, Russia secured record earnings and export levels. As always, in the stock market, prices are not only a reflection of the current demand and offer but also the projected demand and offer in the future. For all those reasons, we found it interesting to explore if a relationship existed between hope/fear of the conflict and the price of gas.

Oil price was also chosen for similar reasons. Oil is another combustible fuel, which can be used to produce electricity. If natural gas is going to become scarce, then oil is going to be one of the most likely substitutes for many applications. Furthermore, the quota controlled by Russia is not big enough to allow them to manipulate the prices in the same way as they do with gas. Considering that the energy crisis could influence the perception of the conflict for European public opinion, it is interesting to also explore the relationship of the oil prices with the proposed hope and fear scores.

One of the very first consequences of Western sanctions on Russia was the fall of the ruble. Many speculations were made on how this would have affected the Russian economy and their ability to repay their debts. The matter became even more interesting when it started to climb back, even reaching higher values than in the pre-conflict period. Since Russia sells a significant part of its gas in rubles, the swinging of the value of ruble is very important to the Russian economy and they are not to be underestimated. The perception of the stability of the country, hence the trust of the market in its currency, could be put in jeopardy by losing this war. This is a good reason to expand the study to the exchange rate between the US dollar and Russian ruble.

The United Kingdom has been one of the most supportive countries of Ukraine since the beginning of the war. Differently from Italy and Germany, they are not part of the European Union, and they have rich reserves of natural gas and oil. United Kingdom Oil and Gas is one of the main stocks for the British energy market. It could prove insightful to understand if there is a relationship between hope and fear toward the Ukrainian war and the stock price of a company that acts in a country involved in the war, is influenced by the price of gas and oil, but has access to national stocks and is less dependent on Russia.

We ran a linear regression analysis between each of these stock market elements and the proposed hope/fear score. Evaluating the results, we conclude that, in terms of the *p*-value, there was no significant correlation between the hope/fear score and Oil-price, Ruble and US dollar exchange rate, and UK Oil-Gas.

**Figure 6:**
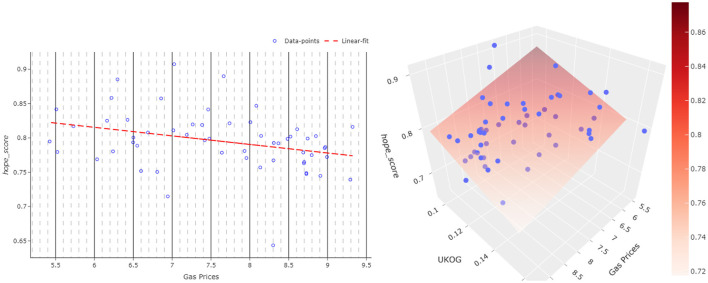
One- and two-parameter regression analysis plots of the hope score. The sub-plot on **(left)** is a scatterplot showing the gas price and the hope score regression analysis. The red dashed line refers to the regression line. The sub-plot on **(right)** shows a 2-parameter regression analysis (UKOG and gas price) in a 3D scatter plot. The surface plotted in this sub-plot shows the 2-regressor model fit plane.

A similar insignificant relationship mentioned previously was also obtained between the fear score and gas prices. However, in terms of the hope score, a significant relationship was found between the hope score and gas prices. To interpret the relationship between the hope score and gas prices, a linear regression was run, having the average daily hope score as the independent variable and the daily closing price as the dependent one. The regression presents a *p*-value of 0.018, showing the significance of the model, whilst a relatively low *R*^2^ value is obtained as 0.1. Furthermore, the Pearson correlation between the two variables is –0.32. As expected, the correlation is negative, so if hope goes up, the gas prices go down, or vice versa (see Figure 6,Left).

We also conducted research on the relationship between all stock variables as regressors and the hope/fear score as the target. Considering a significance threshold value of 0.05 for *p*-value, only the gas and UK Oil-Gas prices returned a significant relationship with the hope score, whilst the fear score does not provide a significant relationship with any of the regressors. Evaluating the results presented in Figure 6, Right, we can conclude that there exists a clear relationship between the hope score and two-regressor model (Gas&OKOG) with an *R*^2^ value of 0.202 and again with a reciprocal proportion.

This analysis shows that the public hope for the result of the conflict is not the primary driver for gas and UKOG prices, but there is indeed a relationship to be explored.

### 4.5. Topic modeling

As described in the previous sections, we now investigate the Reddit data set in terms of topic modeling. To achieve this goal, we utilized R programming language and many different R external packages are used:

- **NLP:** provides the basic classes and methods for natural processing language and poses as a base for the following packages.
- **openNLP:** “an interface to the Apache OpenNLP tools (version 1.5.3). The Apache OpenNLP library is a machine learning-based toolkit for the processing of natural language text written in Java. It supports the most common NLP tasks, such as tokenization, sentence segmentation, part-of-speech tagging, named entity extraction, chunking, parsing, and coreference resolution (The Apache Software Foundation, 2009).”
- **quanteda:** “framework for quantitative text analysis in R. Provides functionality for corpus management, creating and manipulating tokens and ngrams, exploring keywords in context, forming and manipulating sparse matrices of documents by features and feature co-occurrences, analyzing keywords, computing feature similarities and distances, applying content dictionaries, applying supervised and unsupervised machine learning, visually representing text and text analyzes, and more (Benoit et al., 2018).”
- **dplyr:** “is a grammar of data manipulation, providing a consistent set of verbs that help to solve the most common data manipulation challenges (Wickham et al., 2022).”
- **tidytext:** “provides functions and supporting data sets to allow conversion of text to and from tidy formats, and to switch seamlessly between tidy tools and existing text mining packages (Silge and Robinson, 2016).”
- **qdap:** “automates many of the tasks associated with quantitative discourse analysis of transcripts containing discourse. The package provides parsing tools for preparing transcript data, coding tools and analysis tools for a richer understanding of the data Rinker (2020).”
- **plotly** and **ggplot2:** are packages that are used for creating graphics for the analysis.
- **ggthemes:** is a package that enables better aesthetics for graphs.
- **wordcloud:** is a package that allows the creation of word cloud-type graphs.
- **stm:** “The Structural Topic Model (STM) allows researchers to estimate topic models with document-level covariates. The package also includes tools for model selection, visualization, and estimation of topic-covariate regressions (Roberts et al., 2019).” Structural Topic Modeling (STM) is a topic model method. It is a semi-automatic approach that allows us to incorporate metadata, which represents information about each document, into the topic model. STM aims at discovering topics, estimating their relationship to document metadata, and gathering information on how the topics are correlated.

#### 4.5.1. Estimating the optimal number of topics

After the corpus is created, the first step is to extract the diagnostics and estimate the optimal number of topics. Whilst estimating the optimal number of topics, our aim is to maximize two important diagnostics of the *exclusiveness* and *coherence*, whilst keeping *likelihood* high and *residual* diagnostics low enough. Due to the fact that having nine topics would ensure that there would be little mixing up between the topics, a little more importance is given to coherence. On the other hand, data would be very hard to interpret and would be difficult to extract useful information from it.

**Figure 7:**
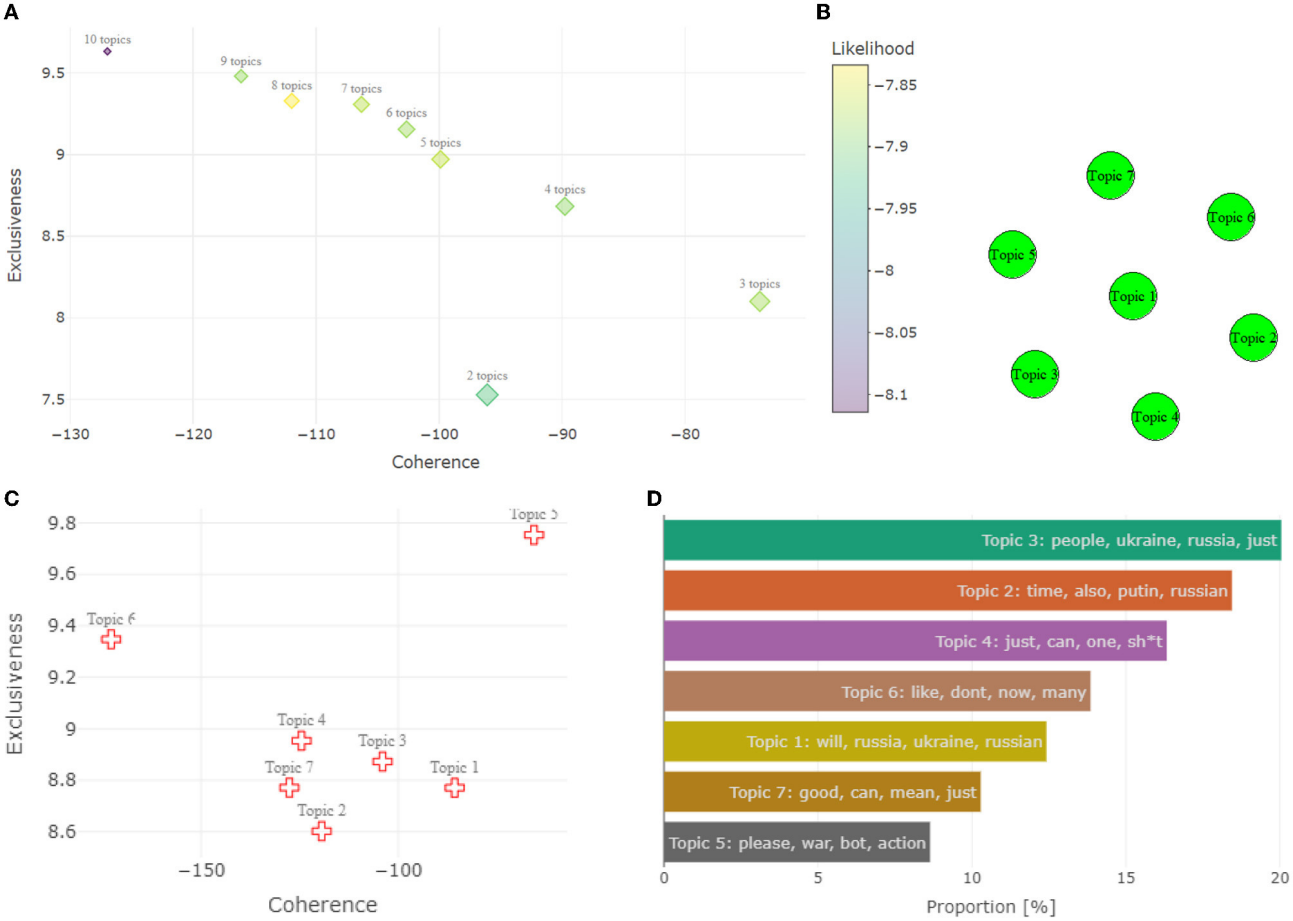
Extensive topic modeling visualizations. **(A)** Top left model selection results with four distinct diagnostics. In addition to the exclusiveness, coherence, and number of topics, the sizes of each marker relate to the residual diagnostic values. **(B)** Top right correlation between topics. **(C)** Bottom left exclusivity and coherence for the individual topics. **(D)** Bottom right topic proportions in the data set.

We present the optimal number of topic selection diagnostic results in Figure 7A. Examining Figure 7A, we can see that Topics 7 and 8 appear to be the optimal choices as a result of the likelihood, residual, and coherence–exclusiveness analysis. We stick to seven topics as the optimal model since it has a lower coherence value compared to eight topics. Thus, seven topics are chosen for this analysis and they can be labeled as:

- Topic 1: Geopolitical arguments
- Topic 2: Russia and government
- Topic 3: Morality of war
- Topic 4: War atrocities
- Topic 5: Submissions in Russian
- Topic 6: Foreign submissions
- Topic 7: Weapons.

Examining Figure 7C, the quality of the topic is investigated in the same way as before, ideally, coherence and exclusivity would be maximized. In this case, it is possible to observe that Topic 5 greatly outperformed all the other topics, especially in coherence. This happens because those observations are all in Russian, which makes them very different from the rest. Topics 1 and 3 score very well on their own in terms of Coherence, whilst Topics 2 and 7 are the worst-performing types overall. Topic 6 on the other side is the one that distinguishes itself the most in terms of exclusiveness, despite having a relatively low semantic coherence. The distribution of the topics is analyzed in Figure 7D. Topic 3 is the most prominent topic, describing around 20% of the database. Topics 5 and 7 are the less popular ones, scoring around 10% each. Considering the correlation analysis plot in Figure 7B, we can clearly conclude that there appears to be no correlation between any of the topics.

#### 4.5.2. Topic 1: Geopolitical arguments

**Figure 8:**
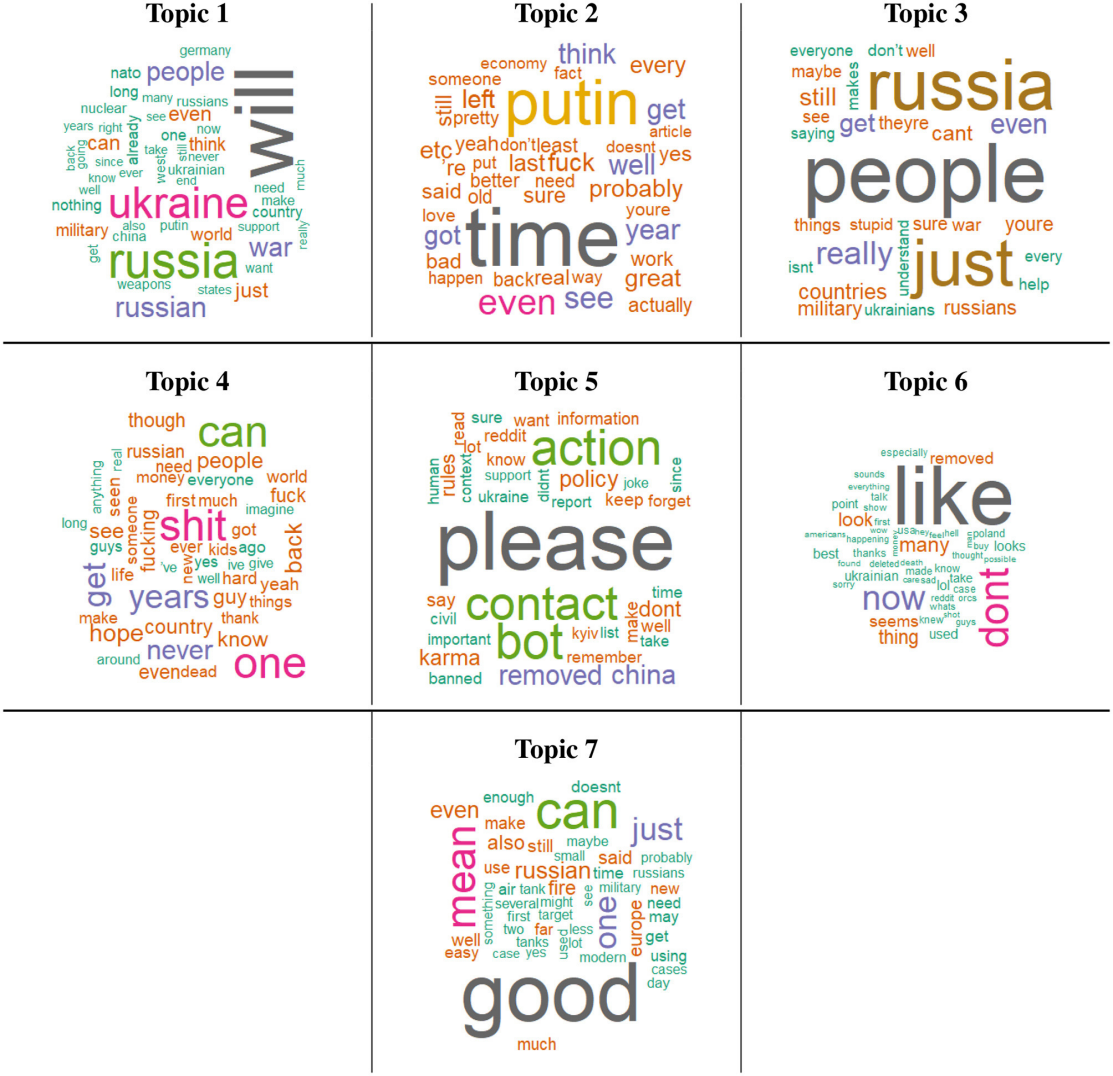
Wordcloud representation for each topic. Each wordcloud highlights some of the specific words (with different sizes depending on the number of submissions) mentioned by the users in their posts.

In Table 2, linear regression modeling results of each topic with hope and fear scores are presented. It can be seen that Topic 1 is positively correlated to both hope and fear. In addition, as shown in Figure 8, Topic 1 is mostly about geopolitical argumentation. The most used words are “Ukraine,” “Russia,” and “will,” showing speculation about the conflict. Other popular words are “NATO,” “China,” “Germany,” “support,” and “sanctions,” a sign of how the broader picture is also depicted in the conversation. Furthermore, “weapons,” “soldiers,” and “nuclear” are also present, demonstrating attention to battles.

**Table 2:** Topic modeling analysis results.

**Results**	**Topic 1**	**Topic 2**	**Topic 3**	**Topic 4**	**Topic 5**	**Topic 6**	**Topic 7**
Intercept	0.1112	0.1943	0.2187	0.1759	0.0800	0.1206	0.0994
*hope*_*score*_	0.0036	–0.0035	–0.0079	0.0056	–0.0040	–0.0091	0.0152
*fear*_*score*_	0.0068	–0.0051	–0.0085	–0.0098	0.0060	0.0137	–0.0031

The correlation to both hope and fear could be explained by the word “will.” If future possibilities are explored, they might be about positive events, hence increasing the hope score, or about scary ones, hence increasing the fear score.

#### 4.5.3. Topic 2: Russia and government

Topic 2 is negatively correlated with both hope and fear. Topic 2 seems to present negative opinions about the Russians and governments. There are many words which refer to them as “Putin,” “Russian,” “Russians,” “government,” “left,” and “right.” Other popular words are “f***,” “bad,” “wrong,” “f***ing,” “old,” and “stop.” As a result, Topic 2 does not lead to clear conclusions due to the low internal coherence of this topic.

#### 4.5.4. Topic 3: Morality of war

Topic 3 is negatively correlated to both hope and fear. Topic 3 seems to be about the moral consequences of the war. Investigating randomly taken submissions as examples shows us that the community discusses (1) the morality of dealing economically with the side of the war, (2) the consequences positive of globalization, and (3) the idea of leaving internal civic debates in Ukraine for later, whilst doing a common front now against the common foe.

These moral considerations are not relevant to hope and fear, for this reason, it is naturally considered that they might score low in both.

#### 4.5.5. Topic 4: War atrocities

Topic 4 is positively correlated with hope, but negatively with fear. Topic 4 is about war atrocities and their devastating effects. Unexpectedly, for this topic, we obtained a positive correlation with hope and a negative one with fear.

#### 4.5.6. Topic 5: Submissions in Russian

Topic 5 is negatively correlated with hope, but positively with fear. Specifically, please note that Topic 5 is composed of submissions in the Russian language. However, the proposed hope dictionary in this article does not accommodate any Russian words in it. This is the potential reason that Topic 5 is negatively correlated to hope. In the case of Fear, we can see that a positive correlation appears here. The potential reason behind this observation might be the usage of some English words in Russian submissions (after checking for several examples of these submissions) which coincide with the words in the Fear dictionary.

#### 4.5.7. Topic 6: Foreign submissions

Topic 6 is negatively correlated to hope but positively correlated to fear. Similarly to Topic 5, Topic 6 is mainly composed of submissions in foreign languages. Most of them will score 0 since their words will not be present in either dictionary. Potentially some similar common words in foreign languages with English created a positive correlation with Fear.

#### 4.5.8. Topic 7: Weapons

Topic 7 is positively correlated with hope, but negatively with fear. Topic 7 is about weapons. Many of the words shown reflect that as given in: “tanks,” “artillery,” “weapon,” “missiles,” “gun,” “range,” “modern,” “expensive,” and “drone.” Others also regard the military in a broader sense, such as “logistic,” “training,” and “equipment.” Finally, “good” is the most used word in the topic.

This explains that the superior Ukrainian equipment reassures the public and increases their hope.

## 5. Conclusions

The results of this study can be seen as the development of a way to measure hope and fear *via* exploiting social media posts of the public all over the world, and an insightful overview of the public opinion on the Russo-Ukrainian conflict, focused predominantly on hope.

The first analysis regards the interest toward the conflict. A steady decline in the number of submissions is observed, whilst the average number of upvotes for the posts does not increase or decrease. This shows a relative loss of interest, due to the stagnation of the news. In fact, the analysis takes place mostly during “phase two” of the war, characterized by a slow but certain Russian advance. On the other side, the average number of upvotes remains constant, demonstrating that the potential interest is still present. The public is still there, however it just needs something new to get engaged with and participate more actively again.

The second analysis is about hope. Following the events of the war, hope strongly decreases after the symbolic and strategic losses of Azovstal (Mariupol) and Severodonetsk. After that, it stabilizes in its slow decrease, mirroring the tides of phase two of the conflict. Spikes in hope, both positives and negatives, are present after important battles, but also some non-military events, such as Eurovision and football games. This is an interesting insight because it shows how morale is not only formed by the objective results of the war, but also by emotional events.

The following analysis pertains to fear. Its trend is stable during the entire analysis, meaning that the tides of the war itself did not influence it significantly. There is a minor negative correlation with hope. It is interesting to notice that they are not inversely correlated. This means that hope and fear could coexist in public opinion in specific instances.

Furthermore, the popularity of the two countries and their leaders is analyzed using a polarity score. The most obvious consideration is that Zelenskyy and Ukraine constantly outperform Putin and Russia. Despite being relatively volatile, the trend seems to remain constant. A key takeaway from this development is that a strong opinion is formed, and without serious upheavals, it will not change.

Moreover, the relationship between fear/hope and relevant financial items was explored. A significant relationship (which is negative) between hope and the gas price was found. With the increase in hope, gas prices would decrease, or vice versa. A reason for that development could be that there is hope that a Ukrainian victory in the war would put at ease again the gas flow from Russia to Europe. Since this aspect has been selected as the fundamental analysis *via* a limited amount of information, more studies would need to be done to fully explore this relationship.

Finally, the topic modeling of the developed data set is studied. The submissions in the English language are about five different topics: geopolitical arguments, Russia and government, the morality of war, war atrocities, and weapons. These are the topics which have caught the public eye the most in the analyzed period. Geopolitical arguments are positively correlated with both hope and fear. The morality of war, Russia, and government is negatively correlated with both hope and fear. Discussions about weapons are positively related to hope and negatively to fear, and surprisingly, the same applies to war atrocities.

## Data availability statement

The raw data supporting the conclusions of this article will be made available by the authors, without undue reservation.

## Ethics statement

Ethical approval was not required for the study involving human data in accordance with the local legislation and institutional requirements. The social media data was accessed and analyzed using the official Reddit API.

## Author contributions

AG and OK wrote the main manuscript text and created data visualization outputs, analyzed the results, and reviewed the manuscript. AG created the web scraping script and collected the data and conducted evaluation and validation experiment(s). OK supervised the research. All authors contributed to the article and approved the submitted version.

## 6. Appendix

### 6.1. Table explaining all developed data set

We present Table A1 to clearly show all the developed data sets for this paper. Data set 0 is basically the main data set which is daily scraped from Reddit.com. It is then used for further analysis in Section 4, and 10 different versions of this data set have been created.

**Table A1:** All the data sets created for applications in Section 4.

**#**	**Description**	**# Samples**	**# Features**	**Test case**
Data set 0	Data set containing all the submissions in the time interval	945778	14	All
Data Set 1	Daily data set	81	14	4.1 & 4.2
Data Set 2	Zelenskyy data set	4255	3	4.3
Data Set 3	Putin data set	30663	3	4.3
Data Set 4	Ukraine data set	89736	3	4.3
Data Set 5	Russia data set	137419	3	4.3
Data Set 6	Ruble data set	56	5	4.4
Data Set 7	Oil Prices data set	56	5	4.4
Data Set 8	Gas prices data set	56	5	4.4
Data Set 9	UKOG data set	56	5	4.4
Data Set 10	Hope-Fear Stock data set	56	7	4.4
